# iASPP facilitates tumor growth by promoting mTOR-dependent autophagy in human non-small-cell lung cancer

**DOI:** 10.1038/cddis.2017.515

**Published:** 2017-10-26

**Authors:** Yijun Xue, Haibo Han, Lina Wu, Bo Pan, Bin Dong, C Cameron Yin, Zhihua Tian, Xijuan Liu, Yue Yang, Hong Zhang, Yingyu Chen, Jinfeng Chen

**Affiliations:** 1 Key laboratory of Carcinogenesis and Translational Research (Ministry of Education/Beijing), Department of Thoracic Surgery II, Peking University Cancer Hospital & Institute, Beijing, China; 2 SDIC Microalgae Biotechnology Center, China Electronics Engineering Design Institute, Beijing, China; 3 Key laboratory of Carcinogenesis and Translational Research (Ministry of Education/Beijing), Department of Biobank, Peking University Cancer Hospital & Institute, Beijing, China; 4 Key laboratory of Carcinogenesis and Translational Research (Ministry of Education/Beijing), Department of Central Laboratory, Peking University Cancer Hospital & Institute, Beijing, China; 5 Department of Hematopathology, The University of Texas MD Anderson Cancer Center, Houston, TX, USA; 6 Key Laboratory of Medical Immunology, Ministry of Health, Peking University Health Science Center, Beijing, China; 7 Peking University Center for Human Disease Genomics, Peking University, Beijing, China

## Abstract

Autophagy serves a critical function in the pathogenesis, response to therapy and clinical outcome in cancers. Although a recent report showed a role of iASPP in suppressing autophagy, its potential activity as a regulator of autophagy has not been investigated in lung cancer. Here we investigated the potential function and molecular mechanism of iASPP in mediating autophagy in human non-small-cell lung cancer. Our data suggested that forced expression of iASPP triggered autophagic flux, while inhibition of iASPP suppressed autophagy at the autophagsome formation stage *in vitro*. Furthermore, *in vivo* overexpression of iASPP in SCID/NOD mice promoted tumorigenesis and autophagy, with an increase in the conversion from LC3-I to LC3-II. The effects of iASPP were mediated through activation of mTOR pathway. Finally, cytoplasmic iASPP expression was upregulated in lung cancer patients, and was identified as an independent poor prognostic factor for lung cancer-specific death in patient samples. Taken together, our data showed that iASPP could promote tumor growth by increasing autophagic flux, and iASPP could serve as a poor prognostic factor and a potential therapeutic target in lung cancer.

Non-small-cell lung cancer (NSCLC) accounts for ~85–90% of all lung cancers, which remains the leading cause of cancer-related death worldwide.^1^ Given its high prevalence and poor 5-year survival rate of 18% in all stages,^1^ it is important to detect new prognostic and predictive markers, as well as novel and more effective treatment options.

The apoptosis-stimulating protein of p53 family (ASPP) stimulates the apoptotic function of p53 upon DNA damage and functions as tumor suppressors. ASPP1 and ASPP2, two members of the ASPP family, can bind to p53 protein and aid the transactivation function of p53 on pro-apoptotic genes, thereby modifying p53-dependent apoptosis responses.^2^ In addition, they can also enhance the p53-independent apoptosis by binding to two other members of the family, p63 and p73, without altering the cell cycle.^3^ The inhibitor of ASPP (iASPP, encoded by *PPP1R13L*) functions as oncogene by binding to apoptosis-regulating proteins such as p53, p63, p73, Bcl-2, NF-*κ*B, p65 and so on, resulting in an inhibition of apoptotic cell death typically after DNA damage.^2^ Aberrant expression of ASPP1, ASPP2 and iASPP has been detected in many kinds of cancer cells. High expression of iASPP was identified to facilitate prostate cancer progression,^4^ resist chemoradiotherapy^5^ and be associate with worse disease status and poor survival.^6, 7^

One recent study for the first time reported that iASPP acts as an autophagy inhibitor in non-transformed keratinocytes.^8^ However, little is known about whether and how iASPP affects autophagy activity in cancer cells. As an emerging hallmark of cancer, aberrant autophagy, serves a critical function in the pathogenesis, outcome and response to therapy in a number of cancers.^9^ The role of autophagy in cancer has been controversial, with some reports indicating that autophagy suppresses tumor development, whereas other reports suggesting that autophagy contributes to tumor growth and progression by attenuating cellular metabolic stress and resisting therapeutic agent-induced cell death.^10^ A better understanding of the molecular mechanism and regulation of autophagy may provide novel therapeutic targets.

Here we explored the role of iASPP in lung cancer. We showed that expression of iASPP was important for modulating autophagy metabolic process in lung cancer cells. In addition, silencing iASPP expression could not only promote the apoptotic signaling but also prevent autophagy by activating mammalian target of rapamycin (mTOR) and promoting autophagosome formation. Moreover, high cytoplasmic expression of iASPP was an independent prognostic factor and was associated with poor survival of patients with NSCLC. Taken together, our results indicate that iASPP may serve as a poor prognostic factor and a potential therapeutic target in NSCLC.

## Results

### Forced expression of iASPP increases cellular autophagy in H1975 cells

**Figure 1:**
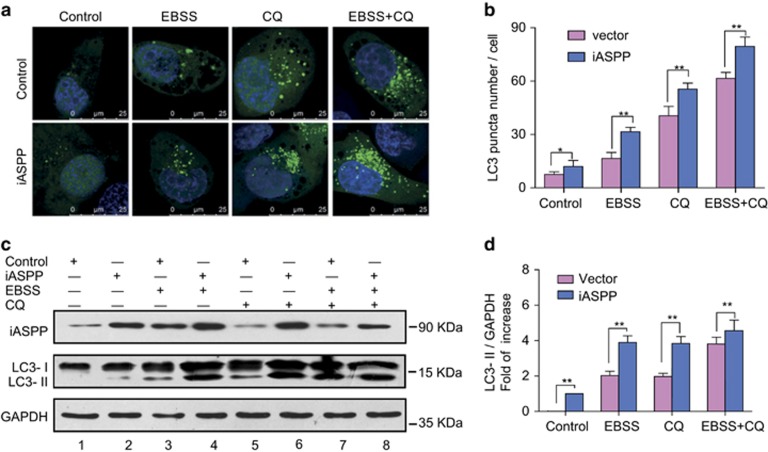
Ectopic expression of iASPP induces autophagy in H1975 cell. (**a**) Endogenous levels for LC3 (green fluorescence) was visualized on confocal microscope by immunofluorescenc staining. (**b**) LC3 dots in control or iASPP-overexpressing cells treated with autophagy inducer as in (**a**) were counted. Data represent mean±S.E.M. of at least 100 cells scored (*P<0.05, **P<0.001). (**c**) Conversions of LC3-I to LC3-II in cells treated as in (**a**) were determined by western blotting. GAPDH was a loading control. (**d**) The ratio of endogenous LC3-II to LC3-I protein level treated as in **c** was counted. The value in the iASPP-overexpressing cells without any treatment was normalized as 1

To select appropriate cell lines for iASPP knockdown and iASPP overexpression, we first analyzed the iASPP expression in seven NSCLC cell lines. As shown in Supplementary Figure S1A, among the seven cell lines, H1975 displayed the lowest iASPP expression. Then, we decide to overexpress iASPP in the H1975 cells. Autophagy has pro- or anti-survival effects on cancer cells depending on tumor type or context. To evaluated whether autophagy was induced by iASPP in lung cancer cells, we analyzed the localization and distribution of endogenous LC3 in lung cancer cells by immunofluorescence. We found an increase of LC3 dots, a measure for autophagy, in iASPP-overexpressed H1975 cells compared with the diffuse pattern in control cells (Figures 1a and b, left). Autophagy inducer, Earle’s balanced salt solution (EBSS) alone, or late-phase autophagy inhibitor (also cause an increase in LC3-II levels) chloroquine (CQ) alone, or EBSS together with CQ also caused accumulation of the LC3 dots in cells, but the abundance of LC3 dots in iASPP-overexpressed cells was much more than that in the control cells (Figures 1a and b). Furthermore, we monitored the alteration of autophagic flux mediated by iASPP overexpression using immunoblot-based LC3 flux assay. The expression level of LC3-II (the cleaved and lipidated form of microtubule-associated protein 1 light chain 3), an early marker for the formation of autophagosomes, was analyzed. The conversion of LC3-I to LC3-II was augmented in the iASPP-overexpressing cells, but not in the control cells (Figure 1c, middle panel, lane 2 *versus* lane 1 and Figure 1d). EBSS alone, CQ alone or EBSS together with CQ caused accumulation of LC3-II in both of the iASPP-overexpressing and the control cells, but the increase was much more significant in the former (Figure 1c middle panel, lane 4 *versus* lane 3, lane 6 *versus* lane 5 and lane 8 *versus* lane 7, and Figure 1d).

### Knockdown of iASPP suppresses cellular autophagy

**Figure 2:**
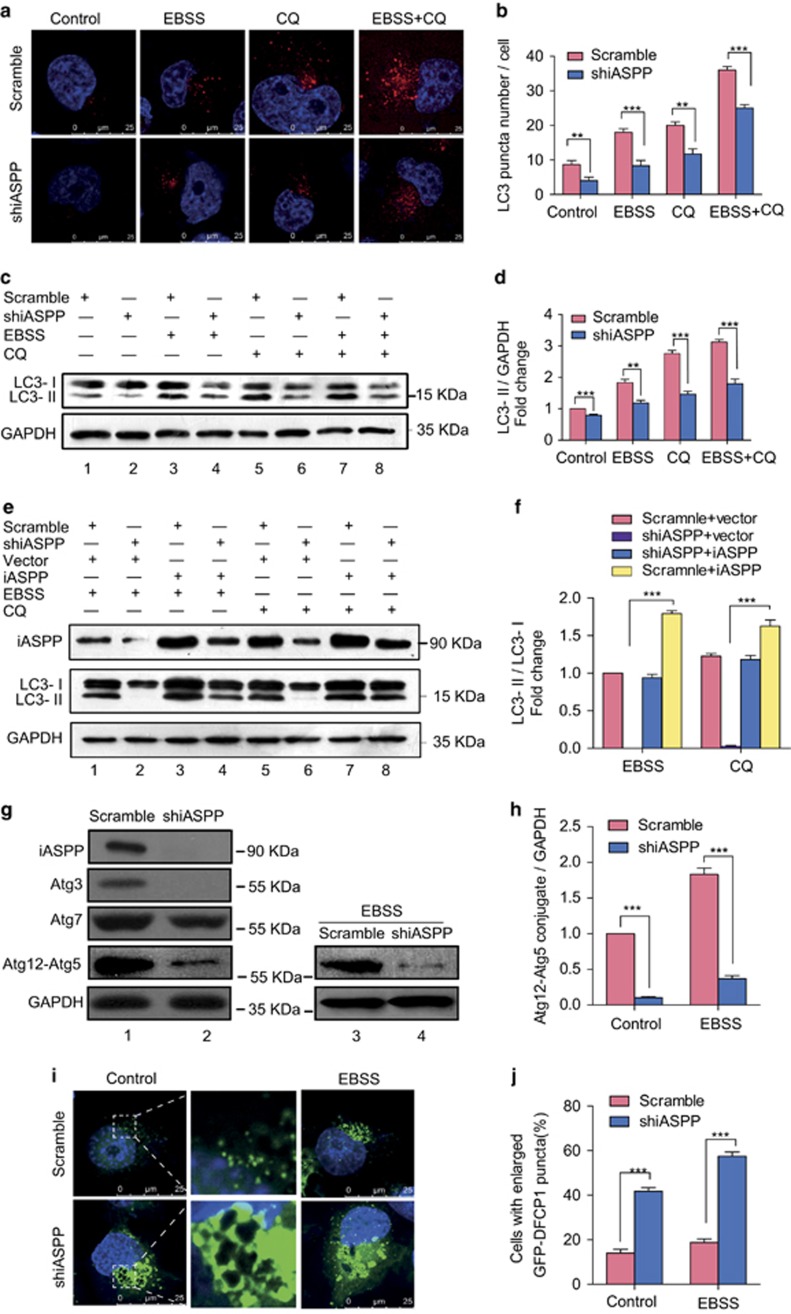
Knockdown of iASPP impairs cellular autophagy by suppressing the stage of autophagosome formation in A549 cells. (**a**) Endogenous levels for LC3 (red fluorescence) was visualized on confocal microscope by immune fluorescence staining. (**b**) LC3 dots in scramble control or shiASPP cells treated with autophagy inducer as in **a** were counted. Data represent mean±S.E.M. of at least 100 cells scored (**P<0.01, ***P<0.001). (**c**) Conversions of LC3-I to LC3-II in cells treated as in (**a**) were determined by western blotting. GAPDH was a loading control. (**d**) The ratio of endogenous LC3-II to LC3-I protein level treated as in **c** was counted. The value in the scramble control cells without any treatment was normalized as 1. (**e**) A549 cells were co-transfected with Scramble vector (or shiASPP) and vector (or iASPP) as indicated for 24 h, and then conversions of LC3-I to LC3-II were measured by western blot. (**f**) Qualification of iASPP protein level and LC3-II to LC3-I protein level as indicated in (**e**). (**g**) Western blot analysis of autophagy-related proteins in A549 cells transfected with scramble or shiASPP vectors for 24 h. (**h**) Quantification of Atg12–Atg5 conjugates relative to GAPDH. The average value in the cells transfected with scramble vector was normalized as 1. (**i**) Localization of GFP-DFCP1 puncta (green). Nuclei were stained with H33342. (**j**) Percentages of cells with abnormal enlarged GFP-DFCP1 puncta were quantified. Data in (**b**, **d**, **f**, **h** and **j**) are the means±S.E.M. of results from three independent experiments (**P<0.001, ***P<0.001)

To further assess the role of iASPP in autophagy, we knocked down iASPP expression by short-hairpin RNA (shRNA) in A549 cells. The expression of iASPP was inhibited efficiently by shiASPP (Figure 2g, upper panel). Immunofluorescence study showed that knockdown of iASPP led to a decrease in LC3 dots as compared with scramble cells (Figures 2a and b, left). In addition, the accumulation of LC3 dots in iASPP-knockdown cells resulted from EBSS, CQ or EBSS together with CQ, was much less than that in scramble cells (Figures 2a and b). The cytosolic form of LC3 (LC3-I, 18 kDa) and the cleaved, lipidated and autophagosome-bound form (LC3-II, 16 kDa) was identified by western blot. Compared with scramble cells, the conversion of LC3-I to LC3-II was abrogated after iASPP knockdown in all types of cells including those with no treatment, treated with EBSS alone, treated with CQ alone, or treated with EBSS and CQ (Figure 2c, lane 2 *versus* lane 1, lane 4 *versus* lane 3, lane 6 *versus* lane 5 and lane 8 *versus* lane 7, and Figure 2d). Moreover, we also knocked down iASPP expression by shRNA in additional H1299 cells with a relative high iASPP expression. As indicated in Supplementary Figure S1B, consistent with the result above, compared with scramble cells, the conversion of LC3-I to LC3-II also decreased after iASPP knockdown in different treatment including no treatment, EBSS treatment alone, CQ alone, or EBSS and CQ by western blot analysis. These results suggested that knockdown of iASPP impaired cell autophagy in A549 and H1299 cells.

We then performed a rescue assay by co-transfecting iASPP expression plasmid with iASPP shRNA followed by EBSS treatment or CQ treatment. Western blot analysis showed that the conversion of endogenous LC3-I to LC3-II was significantly increased in cells co-transfected with iASPP expression plasmid and iASPP shRNA compared with those cells transfected with iASPP shRNA alone (Figure 2e, middle panel, lane 4 *versus* lane 2 and lane 8 *versus* lane 6, and Figure 2f), suggesting that rescue iASPP partially reversed the phenotype initiated by shiASPP. Furthermore, we assessed the protein level of autophagy-associated genes (Atg) by western blot. The iASPP-silenced cells expressed lower levels of the autophagy-associated proteins, including Atg3, Atg7 and Atg12–Atg5 conjugate, than the scramble control cells (Figure 2g, lane 2 *versus* lane 1, and 2h, left). Compared with control cells treated with EBSS, the protein level of conjugated Atg12–Atg5, which is essential for autophagosome formation, was also decreased in iASPP-silenced cells treated with EBSS (Figure 2g, lane 4 *versus* lane 3, and 2h, right). These data show that iASPP rescued the shiASPP-induced inhibition of autophagy, and suggest that endogenous iASPP has an essential role in cellular autophagy.

### Knockdown of iASPP impairs autophagosome formation

To further investigate the stage at which the autophagy process is inhibited in iASPP-silenced cells, we tested omegasome formation, also known as the phagophore assembly site, which can be labeled by exogenous GFP-DFCP1. A549 cells were co-transfected with GFP-DFCP1 and shiASPP or scramble vectors, and then observed under a fluorescence microscope. Compared with scramble cells, shiASPP-transfected cells displayed abnormally enlarged GFP-DFCP1 puncta (Figure 2i, lower panel), and the percentage of this kind of cells in the iASPP-silenced cells was much higher than that in the scramble control cells treated with or without EBSS (Figures 2i and j, ***P<0.001), suggesting the accumulation of omegasomes. Collectively, these results indicated that iASPP might regulate the autophagy at the stage of autophagosome formation.

### Inhibition of autophagy induced by shiASPP is dependent on activation of mTOR pathway

**Figure 3:**
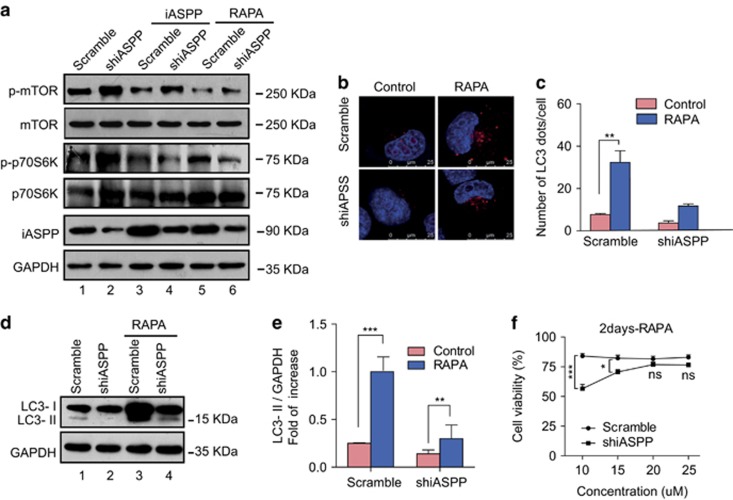
shiASPP-induced autophagy inhibition is dependent on mTOR activation in A549 cells. (**a**) Western blot analysis of mTOR pathway proteins in scramble or shiASPP cells, either with iASPP rescue or with rapamycin treatment. (**b**) Endogenous levels for LC3 (red fluorescence) were visualized on confocal microscope by immunofluorescenc staining. (**c**) LC3 dots in scramble control or shiASPP cells treated with mTOR inhibitor as in (**b**) were counted. Data in (**c**) represent mean±S.E.M. of at least 100 cells scored (**P<0.01). (**d**) Conversions of LC3-I to LC3-II in cells treated with rapamycin were determined by western blotting. GAPDH was a loading control. (**e**) The ratio of endogenous LC3-II to LC3-I protein level treated as in (**d**) was counted. The value in the scramble control cells with rapamycin treatment was normalized as 1. (**f**) Cells were treated with rapamycin at different doses, and their viability was determined by the CCK8 assay (*P<0.05; ***P<0.001; NS, no significance)

To gain insight into how iASPP knockdown alters the autophagy of A549 cells, we examined the expression of intracellular signaling molecules using a PathScan Intracellular Signaling Array Kit (Cell signaling Technology, Danvers, MA, USA). As shown in Supplementary Figure S1C, compared with the scramble cells, iASPP knockdown obviously upregulated the phosphorylated levels of mTOR (Ser-2448). Consistent with the above results, western blot analysis also demonstrated an increase in mTOR signal pathway proteins (p-mTOR and p-p70S6K; Figure 3a, lane 2 *versus* lane 1) in iASPP-silencing cells. To further determine whether mTOR, the major negative regulator, contributed to autophagy inhibition in iASPP-silencing cells, we performed a rescue experiment and inhibited mTOR pathway using rapamycin, a mTOR inhibitor. Compared with scramble control cells, both iASPP rescue and rapamycin treatment reversed the effect of shiASPP on p-mTOR and p-p70S6K (Figure 3a, lane 4 *versus* lane 3 and lane 6 *versus* lane 5). In addition, rapamycin restored both LC3 puncta (Figures 3b and c) and the conversion of LC3-I to LC3-II (Figures 3d and e) in iASPP-silenced cells. Furthermore, we have shown previously that knockdown of iASPP expression resulted in decreased cell proliferation and colony formation in lung cancer cells *in vitro*, and here we found that rapamycin also restored the anti-viability effect of shiASPP in a dose-dependent manner at 48 h (Figure 3f). Taken together, these data suggest that autophagy inhibition resulting from silencing of iASPP is mainly dependent on mTOR activation.

### iASPP promotes cell proliferation and tumor growth in xenograft tumor model

**Figure 4:**
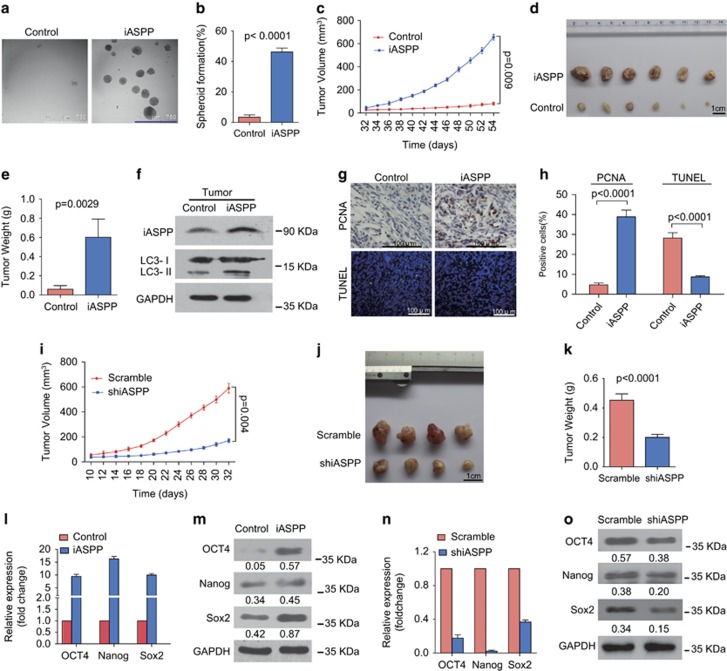
iASPP supported cell proliferation and tumor growth. (**a**) The morphology of cells presented at spheroid formation after 14 days. Scale bars: 750 *μ*m. (**b**) The number of spheriods larger than 50 *μ*m was counted under a dissecting microscope. The data represented the mean spheroid formation rate±S.E.M. of cells from three independent experiments. (**c**–**e**) Tumors formed from H1975 cells in xenografts of NOD/SCID mice were measured, separated and weighed up. (**f**) The protein expression levels of iASPP and conversion of LC3-I to LC3-II in mixture of six tumors for each group were determined by western blotting. GAPDH was used as a loading control. (**g**) Immunohistochemical staining of PCNA protein for measurement of proliferation and immunofluorescence staining for measurement of apoptosis by TUNEL assay in tumor xenografts. (**h**) Quantification of PCNA-positive or TUNEL-positive cells as the mean±S.E.M. percentage of positive cells per field from six random microscopic fields. (**i**–**k**) Tumors formed from A549 cells in xenografts of NOD/SCID mice were measured, separated and weighed up. Tumor volume was calculated using the formula: *V*=length × width^2^/2. The curve in **c** and **i** represented the tumor volumes±S.E.M. of six mice for each group on different days. The data in (**e** and **k**) represented the mean tumor weight±S.E.M. for each group. (**l**–**o**) The expression of pluripotency markers, including OCT4, NANOG and SOX2 were performed by qPCR and western blotting. Western blot quantification estimated as relative ratio of each protein to GAPDH were shown under individual blots

In order to reproduce our previous *in vitro* result of the antiproliferative effect of iASPP knockdown in a mouse model, we constructed an iASPP expression plasmid and transfected it into H1975 cell line to establish a stable iASPP-overexpressing cell line. Western blot analysis conformed that iASPP was remarkably overexpressed in H1975 cells (Figure 1c, lane 2 *versus* lane 1). Spheroid formation assay, widely used to detect tumor-initiating cells, was performed to further investigate the effect of iASPP on the ability of self-renew *in vitro*. Compared with the control cells, ectopic expression of iASPP resulted in enlarged spheriod formation and increased cloning efficiency from 3.4 to 46.2% (Figures 4a and b). We then implanted iASPP-expressing H1975 cells in NOD/SCID mice. As shown in Figures 4c–e, tumors arising from iASPP-expressing cells had significantly increased volume (Figure 4c, *P*<0.05) in a time-dependent manner and weight (Figures 4d and e, *P*<0.05), when compared with the control group. We also assessed conversion of LC3-I to LC3-II in the tumors by western blot. As shown in Figure 4f, tumors in the iASPP group showed more conversion of LC3-I to LC3-II compared with the control tumors. To determine whether the increased tumor volume was a consequence of enhanced proliferation or reduced apoptosis, we measured the proliferating cell nuclear antigen (PCNA), a marker of cell proliferation by immunostaining, and detected the apoptosis with *in situ* terminal deoxynucleotidyl transferase (TdT)-dUTP nick-end labeling (TUNEL staining) in tumor xenograft. As shown Figures 4g and h, the PCNA expression in the tumor initiated from iASPP-overexpressing cells was markedly increased compared with tumor formed from scramble cells (Figure 4g, upper panel; and Figure 4h, left). At the same time, there were less apoptosic cells in the iASPP tumors than control tumors (Figure 4g, down panel; and Figure 4h, right). These data suggested that overexpression of iASPP promotes tumor growth by increased cell proliferation and reduced cell apoptosis.

On the other hand, to further confirm our results, we also assessed the effect of iASPP inhibition on tumor growth. Consistent with the above results, tumors derived from cells with iASPP inhibition showed significantly decreased volume (Figure 4i, *P*<0.05) and weight (Figures 4j and k, *P*<0.05), when compared with the scramble group.

Furthermore, we detected the influence of iASPP on the expression level of crucial transcription factors in the maintenance of self-renewal and pluripotency in stem cells, including OCT4, Nanog and SOX2. Clearly, the expression level of OCT4, Nanog and SOX2 in iASPP-overexpressing cells were increased to 9 times, 15 times and 8 times, respectively, as compared with control cells (Figures 4l and m). Consistently, the expression level of these transcription factors in iASPP knockdown cells were significantly decreased (Figures 4n and o). Collectively, iASPP, as an oncogene, can promote cell proliferation and tumor growth partially by activating crucial transcription factors related with stem cells.

### Cytoplasmic iASPP expression is increased in patients with NSCLC and is associated with poor clinical outcome

**Figure 5:**
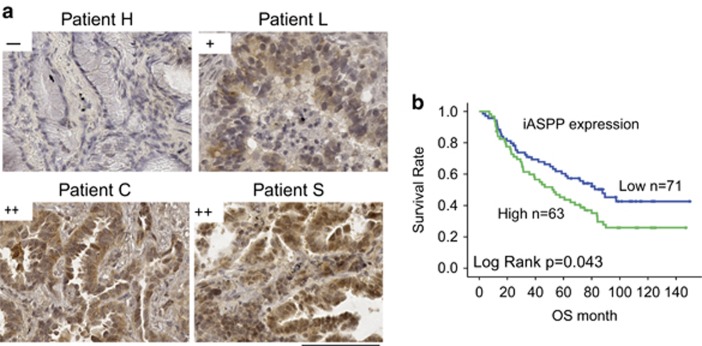
Increased cytoplasmic iASPP expression in lung cancer samples is associated with a poor clinical outcome. (**a**) The expression level of iASPP with immunochemistry staining in human lung cancer samples. (**b**) Increased iASPP expression in lung cancers was associated with increased cancer-specific death after radical surgery following 10 years of follow-up

We assessed the level of iASPP expression in radical resection samples from 134 patients with NSCLC and more than 10 years’ clinical follow-up by immunohistochemistry. Similar to our previous study, an increase in cytoplasmic iASPP expression was observed in NSCLC samples compared with adjacent lung epithelium (Figure 5a and Table 1). Lack of iASSP expression was seen in only 27.6% (37/134) of lung cancer tissues in contrast to 65.7% (88/134) in adjacent non-cancerous tissues (Table 1, Fisher’s exact test for an *R* × *C* table *P*=0.01).

We further investigated whether increased iASPP expression in NSCLC patients has a prognostic significance. Univariate Kaplan–Meier survival analysis revealed that increased cytoplasmic iASPP expression was associated with an increased risk of lung cancer-specific death in patients undergoing radical surgery (Figure 5b). Overall survival time for patients with high cytoplasmic iASPP expression was significantly shorter than those with low cytoplasmic iASPP expression (Figure 5b, median survival time was 53.1 *versus* 87.7 months, *P*=0.043). Furthermore, cytoplasmic iASPP expression in lung cancer patients was identified as an independent prognostic factor for cancer-specific death by Cox regression multivariate analysis (Table 2, HR=1.714, *P*=0.019). These results suggest that increased expression of cytoplasmic iASPP has clinical significance by conferring an increased risk of lung cancer-specific death.

## Discussion

iASPP is an inhibitory member of the ASPP family, which has been reported to be overexpressed as an oncogene in several cancers, such as breast cancer and hepatocellular cancer.^11, 12, 13^ We have shown previously that iASPP was overexpressed in human NSCLC and that downregulation of iASPP inhibited the proliferation and colony formation of lung cancer cells by activating p53/p21/PUMA pathway *in vitro*.^14^ Here we have reinforced the notion of a constitutive cancer-promoting function of iASPP *in vivo*. Moreover, for the first time we showed that iASPP could regulate cell autophagy in lung cancer cells.

Autophagy, a highly conserved catabolic process involving the delivery of cytoplasmic components to lysosomes for degradation, has a pivotal role in the maintenance of the cellular environment. The role of autophagy in cancer has been controversial. It has been shown that iASPP-silenced keratinocytes displayed activation of autophage and protection from apoptosis.^8^ However, our findings suggest that overexpression of iASPP triggered autophagic flux followed by promoting cell growth. Inhibition of iASPP suppressed autophagy at the autophagsome formation stage, as well as cell proliferation. It seems that the function of iASPP in non-transformed cells (such as keratinocytes and primary cultures of lymphocytes and fibroblasts) is totally opposite to that in cancer cells. Non-transformed cells treated with either iASPP-specific siRNA or shRNA reduced levels of apoptosis,^8, 15^ whereas silencing of iASPP in several cancer cell lines promoted apoptosis.^12, 13, 16, 17, 18, 19, 20^ Therefore, unlike keratinocytes, knockdown of iASPP did not induce but rather prevented autophagy in cancer cells. Autophagy has context-dependent functions in different stages of cancers. As a suppressor pathway, autophagy prevents tumor initiation; but as a survival pathway, autophagy contributes to tumor growth and progression by attenuating cellular metabolic stress and resisting therapeutic agent-induced cell death.^10^ Obviously, our data show that autophagy induced by forced expression of iASPP contributed to cancer promotion in NSCLC.

mTOR is a serine/threonine kinase that has an important role in regulating cellular homeostasis and metabolism. The mTOR protein consists of at least two distinct multi-protein complexes, namely mTOR complex 1 (mTORC1) and mTOR complex 2 (mTORC2).^21^ The mTORC1 integrates stimulating signals and then phosphorylates p70 S6 kinase 1 (S6K1) and eukaryotic initiation factor 4E-binding protein 1, which results in cell growth, activation of translation and ribosome biosynthesis. Up to date, mTORC1 is the best-characterized regulator of autophagy. It has been shown that rapamycin and its analogs could inhibit the activity of mTORC1.^22^ For the first time, our study revealed phospho-mTORC1 (Ser-2448) and its downstream effector ribosomal S6 kinase (p-p70S6K) protein were increased after depletion of iASPP, indicating that increased mTORC1 activity might contribute to the anti-autophagic effect of iASPP silencing. Rapamycin treatment could rescue mTOR activation, and at least partially resist anti-autophagy and anti-proliferation effect in shiASPP cells. Furthermore, we observed the accumulation of LC3-labeled structures, enlarged DFCP1 structures and LC3 lipidation-related proteins, including Atg3, Atg7 and Atg12–Atg5 conjugate, in iASPP-silenced cells. Thus, depletion of iASPP function may impair autophagosome formation. Collectively, knockdown of iASPP might block autophagy by mTORC1–P70S6K signaling and then impair autophagosome formation.

Autophagy and apoptosis have significant roles in cell metabolism and survival. However, the interaction between these two processes is still far from being completely understood. Zhou *et al*^23^ found that autophagy postponed apoptotic cell death through Bad–Beclin1 interaction. In our previous study, iASPP depletion suppressed the proliferation of lung cancer cells by stimulating apoptosis through activation of p53/p21/PUMA pathway.^14^ Here our data revealed that higher expression of phosphorylated Bad (Ser112) in shiASPP cells than that in control cells (Supplementary Figure S1C). In addition, increased levels of activated caspase-3, a crucial enzyme in the apoptotic process, and PARP cleavage by caspases, a marker of apoptotic cell death,^21^ were both identified in iASPP-silencing cells, compared with control cells (Supplementary Figure S1D). Taken together, our data indicated that cell apoptosis by iASPP silencing may be associated with autophagy.

Furthermore, we showed that iASPP could activate OCT4, Nanog and SOX2, which are crucial transcription factors in the maintenance of self-renewal and pluripotency in stem cells. To our best knowledge, this is the first report that iASPP could activate the expression of these stem cell markers. These data are in keeping with the reports that autophagy contributes to the enrichment and survival of cancer stem cells.^24, 25^ It has been reported that mTORC1 signaling is essential for the regulation of hematopoietic stem cell quiescence,^26^ but the effect of iASPP on cancer stem cell properties in NSCLC still needs to be further elucidated.

Moreover, we found that iASPP was identified as one of the independent prognosis factors in patients with NSCLC. Activation of autophagy is a hallmark in tumor cells treated with chemotherapy. Autophagy is a protective mechanism of tumor cells induced by stress to avoid low nutrition, ionizing radiation and chemotherapy. Ren *et al*^27^ found that upregulation of autophagy had a major role in cisplatin resistance of lung adenocarcinoma. Here we show that iASPP was upregulated in patients with NSCLC and was associated with increased autophagy and poor clinical outcome. In addition, we found that overexpression of iASPP induced higher levels of expression of stem cell-related transcription factors, suggesting iASPP may have an important role in chemotherapy resistance of lung cancer cells, which may at least partially explain its association with a poor prognosis.

In summary, our results show that iASPP induces autophagy and facilitates tumor growth in lung cancer. iASPP regulates the autophagosome formation through mTOR signaling. We also found that iASPP expression is increased in patients with NSCLC and is associated with poor prognosis. Our findings provide new insights into the function of iASPP and suggest that iASPP may serve as a new therapeutic target for patients with NSCLC.

## Materials and methods

### Cell lines and human tissue samples

Human NSCLC cell lines, A549, H1975 and H1299, were originated from American Type Culture Collection (Manassas, VA, USA) and cultivated in RPMI 1640 medium supplemented with 10% fetal bovine serum, 100 U/ml penicillin and 100 mg/ml streptomycin (Invitrogen, Grand Island, NY, USA) in a humidified atmosphere of 5% CO_2_ at 37 °C.

Lung cancer specimens (*n*=134) and matched adjacent normal tissues were obtained from patients who were treated at Peking University Cancer Hospital from 2000 to 2007. Prior written and informed consent was obtained from all patients or their families. The study was approved by the Research and Ethical Committee of Peking University Cancer Hospital. Clinicopathologic characteristics of the tumors were defined according to the tumor–node–metastasis staging system of the Union for International Cancer Control (Supplementary Table S1). As quality control, we performed Kaplan–Meier survival analysis based on lymph node metastasis status or tumor thrombus, which is commonly considered to be associated with survival of patients with lung cancer. As shown in Supplementary Figure S1E, the median survival of patients with lymph node metastasis or tumor thrombus was significantly shorter than those without lymph node metastasis or tumor thrombus.

### RNA isolation and quantitative real-time PCR

Total RNA was isolated from cells using the Trizol reagent (Invitrogen, Carlsbad, CA, USA). First-strand cDNA was synthesized from total RNA using oligodT_(15)_ primers using moloney murine leukemia virus reverse transcriptase (Invitrogen). Quantitative real-time PCR was performed using SYBR Green PCR Master Mix (Toyobo, Osaka, Japan) on ABI7500 System (Applied Biosystems, Foster City, CA, USA). The expression of the target gene was normalized to that of glyceraldehyde 3-phosphate dehydrogenase (GAPDH). The expression level of target gene was calculated by the 2^−ΔCt^ method, where ΔCt=Ct (target gene)–Ct (GAPDH). The sequences of primers were listed in Supplementary Table S2.

### Plasmid construction and cell transfection

The iASPP-overexpressing vector was purchased from OriGene (RC227121, Rockville, MD, USA) and is named as pCMV-iASPP. Cells were transfected with pCMV-iASPP plasmid followed by 500 mg/ml G418 screening to obtain cells overexpressing iASPP. The lentivirus RNAi vector containing the sequence of shRNA for the iASPP target gene, pGCL-GFP/U6-shiASPP, was purchased from Genchem Company (Shanghai, China). The target sequence of iASPP was 5′-AACACATGGATCTGAAGCAGA-3′, and the sequence of 5′-AATGTACTGCGCGTGGAGA-3′ was used as scramble shRNA control. The lentiviral shuttle vectors were transfected into 293T cells together with lentiviral helper plasmids pHelper1.0 and pHelper2.0 to generate lentiviruses and then infect cancer cells. GFP-LC3 and GFP-DFCP1 plasmids were kindly provided by Professor Yingyu Chen from Peking University Health Science Center. All plasmids were confirmed by sequencing.

### Induction and inhibition of autophagy

Cellular autophagy was induced by nutrient deprivation through incubation in EBSS (Sigma, St. Louis, MO, USA). Autophagy inhibition was achieved by treating cells with 10 *μ*M of CQ (Sigma), a lysosomal inhibitor. Rapamycin (RAPA, a specific mTOR inhibitor, Sigma) was used at 0.1 nM concentration to block mTOR activity.

### Immunohistochemistry and TUNEL staining

Formalin-fixed, paraffin-embedded tissue sections were de-paraffined and rehydrated using graded alcohols. Endogenous peroxidase activity was inactivated by 3% H_2_O_2_. Antigen retrieval was performed using boiling citric acid buffers. Sections were blocked with 5% non-fat milk and incubated with primary antibody (Anti-iASPP: Rockland Immunochemicals, Philadelphia, PA, USA; Anti-PCNA: Abcam, Cambridge, UK) at 4 °C overnight. A horseradish peroxidase (HRP)-labeled secondary antibody was added for 60 min followed by a 3,3′-diaminobenzidine solution before counterstained with hematoxylin. Sections were dehydrated and photographed with Aperio Versa scanner (Leica, Welzler, Germany). Stained slides were scored according to the intensity of staining (−: 0; +: 1; ++: 2; and +++: 3) and the percentage of cells staining positive for each antigen (<5%: 0; 5–24%: 1; 25–49%: 2; 50–74%: 3; and ≥75%: 4) by two independent pathologists. The iASPP expression score of each slide was calculated by the formula: score=the score of staining intensity × the score of percentage of positive cells. The immunohistochemistry result was defined as − (score: 0–1), + (score: 2–4), ++ (score: 5–8) and +++ (score: 9–12). For proliferation analysis, the percentage of PCNA-positive cells were calculated. TUNEL staining was performed by *In Situ* Cell Death Detection Kit (Sigma-Aldrich, Milwaukee, WI, USA) according to the manufacturer’s protocol. Briefly, deparaffinized and hydrated sections was pretreated using 10 *μ*g/ml proteinase K digestion for 20 min, and incubated in TdT reaction mixture for 1 h at 37–40 °C in humidified chamber followed by wash three times in PBS for 5 min with the counterstain DAPI (4′, 6-diamidino-2-phenylindole) for 10 min. Fluorescence was photographed with microscope (Leica) and the percentage of TUNEL-positive cells was determined.

### Spheroid formation assay

For tumor sphere formation, 100 cells/well were plated in serum-free DMEM/F12 medium (Invitrogen), supplemented with 2% B27 (Invitrogen), 20 ng/ml bFGF (Peprotech, Rocky Hill, NJ, USA), 10 ng/ml HGF (Peprotech) and 1% methylcellulose (Sigma) into Ultra-low attachment 96-well plate (Corning, Corning, NY, USA) for 14 days. Individual spheres ≥50 *μ*m in each replicate well were counted under a microscope (Olympus, Shinjuku, Japan).

### Tumorigenicity assay

For assessment of tumor formation, 10^5^ live cells in 200 *μ*l Matrigel/PBS (1:1) were injected subcutaneously into the back of 6-week-old NOD/SCID male mice (NOD.CB17-prkdcscid/NcrCrl, Vitalriver, Beijing, China). The tumor growth was monitored by electronic calipers and tumor volume was counted by the formula: volume=length × width^2^/2. All animal studies were approved by the institutional guidelines of Peking University Cancer Hospital Animal Care Committee.

### Immunofluorescence

Cells treated with different reagents were seeded on glass coverslips and grown to confluence. After 24 h treatment, cells were fixed with formaldehyde for 5 min, permeabilized in 0.5% Triton X-100 for 10 min and then incubated with primary antibody at 37 °C for 1 h. To observe the endogenous LC3 puncta, cells were exposed to LC3 antibody (Sigma). An appropriate fluorescence-labeled secondary antibody with DAPI (1 *μ*g/ml) was then added and incubated at 37 °C for 1 h. Cells transfected with GFP-DFCP1 plasmids were directly observed by fluorescence microscopy. The number of LC3 puncta per cell and the percentage of enlarged GFP-DFCP1 structures were counted in five non-overlapping fields in three independent experiments. Glass coverslips were mounted in 90% glycerol medium and photographed with laser scanning confocal microscope (Leica).

### Western blot analysis

Cells were lysed with RIPA buffer containing protease inhibitor cocktail (Roche, Basel, Switzerland). Protein concentration was assessed with a Bio-Rad Protein Assay Kit (Bio-Rad Laboratories, Hercules, CA, USA). Equal amounts of proteins from each sample (30 *μ*g) were separated by 10 or 12% SDS-PAGE and transferred to polyvinylidene difluoride membrane (Merck Millipore, Billerica, MA, USA). The membrane was blocked for 1 h in PBS containing 5% non-fat milk. For detection with primary antibodies (anti-iASPP: Rockland; anti-GAPDH: Santa Cruz Biotechnology, Dallas, TX, USA; anti-p-mTOR and anti-ATG3: Cell Signaling Technology, Danvers, MA, USA; anti-Beclin1 and anti-ATG7: Abcam; anti-LC3B, anti-GFP, anti- mTOR, anti- p70S6K and anti-p-p70S6K: Sigma), the membranes were incubated with the appropriate antibody at 4 °C overnight, followed by incubation with HRP-conjugated secondary antibodies, and then developed using ECL western blot detection reagents (PerkinElmer, Waltham, MA, USA).

### Cell viability assay

In all, 5 × 10^3^ cells/well were seeded in 96-well plates containing 100 *μ*l of culture medium. After rapamycin treatment, 10 *μ*l of solution from Cell Counting Kit-8 (Dojindo Molecular Technologies, Kumamoto, Japan) was added to each well. These plates were continuously incubated for 2 h in a humidified CO_2_ incubator at 37 °C and then the intensity was measured at a wavelength of 450 nm. Absorbance values were presented as the mean±S.E.M. of the mean value from three independent experiments for each treatment. The absorbance of untreated cells was considered to be 100%.

### Statistical analysis

For continuous variables, data were presented as mean±S.E.M. of the mean value from three independent experiments and analyzed by the Student’s *t*-tests to determine the difference between two independent groups. Otherwise, data were analyzed using methods as indicated in the text. A two-sided *P*-value of 0.05 was considered to be statistically significant. All statistical data were performed by SPSS 13.0 (SPSS Inc., Chicago, IL, USA).

## Statistical analysis

Publisher’s Note: Springer Nature remains neutral with regard to jurisdictional claims in published maps and institutional affiliations.

## Figures and Tables

**Table 1:** The expression pattern of iASPP in lung cancer samples revealed in immunohistochemistry analysis

	**Adjacent tissues**				
**Tumor tissues**	**(−)**	**(+)**	**(++)**	**(+++)**	**Sum**
Negative (−)	28	4	5	0	37 (27.6%)
Weak positive (+)	19	11	3	0	33 (24.6%)
Positive (++)	40	4	16	1	61 (45.5%)
Strong positive (+++)	1	1	1	0	3 (2.2%)
Sum	88 (65.7%)	20 (14.9%)	25 (18.7%)	1 (0.7%)	134

Fisher’s exact test for an R × C table *P*=0.01

**Table 2:** Multivariate analysis of clinical prognostic factors for lung cancer-specific death in patients undergoing radical surgery in tissue microarray cohort

	**Lung cancer death (*n*=134)**	
	**HR (95% CI)**	***P***
iASPP staining	1.714 (1.092–2.691)	0.019
TNM stage	1.491 (1.166–1.906)	0.001
Pathological types	0.919 (0.665–1.270)	0.609
Differentiation grade	1.098 (0.895–1.348)	0.370

Abbreviations: CI, confidence interval; HR, hazard ratio
