# Supplementary figures and images for: iASPP facilitates tumor growth by promoting mTOR-dependent autophagy in human non-small-cell lung cancer
Source: Cell Death Dis. 2017 Oct 26;8(10):e3150–. doi: 10.1038/cddis.2017.515 (PMC5682680; doi:10.1038/cddis.2017.515)

A

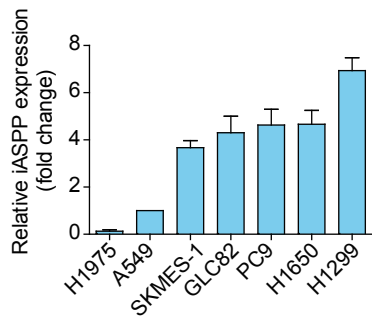

B

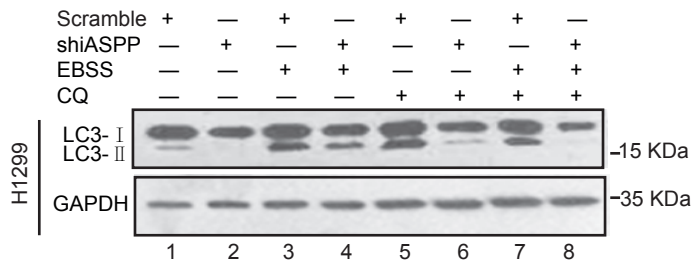

C

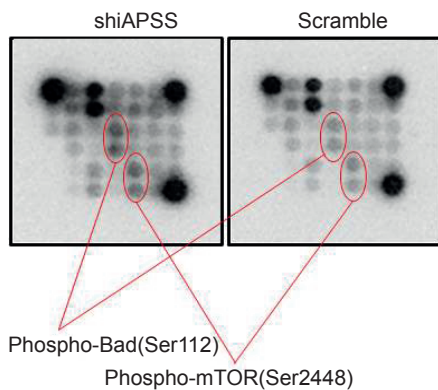

D

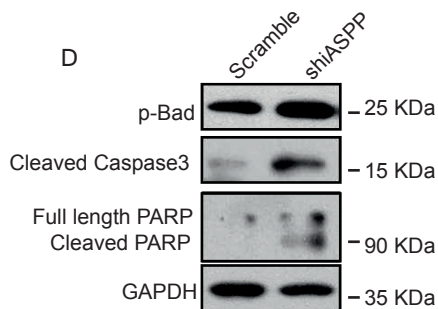

E

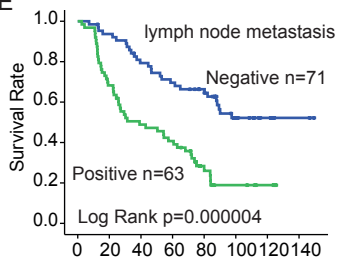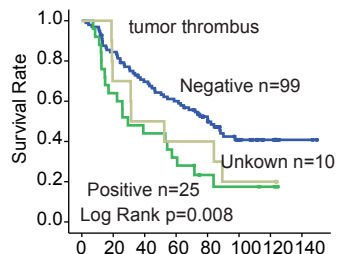

Supplement: Supplementary Figures [file cddis2017515x1.pdf]
