# Supplementary material for: iASPP facilitates tumor growth by promoting mTOR-dependent autophagy in human non-small-cell lung cancer
Source: Cell Death Dis. 2017 Oct 26;8(10):e3150–. doi: 10.1038/cddis.2017.515 (PMC5682680; doi:10.1038/cddis.2017.515)
Supplement: Supplementary Table 1 [file cddis2017515x2.docx]

Table S1 Clinical characteristics of the patients undergoing radical surgery in tissue microarray cohort

|  |  | Radical surgery (n=134) |
| --- | --- | --- |
| Median age (range) | | 60 years(range 39-77) |
| TNM stage | Ⅰ | 50(37.3%) |
|  | Ⅱ | 26(19.4%) |
|  | Ⅲ | 58(43.3%) |
| Pathological types | Squamous cell carcinoma | 55(40.0%) |
|  | Adenocarcinoma | 67(50.0%) |
|  | Carcinoid | 3(2.2%) |
|  | Large cell carcinoma | 4(3.0%) |
|  | Adenosquamous carcinoma | 5(3.7%) |
| Differentiation grade | Undifferentiation | 3(2.2%) |
|  | low grade | 20(14.9%) |
|  | Intermediate grade | 76(56.8%) |
|  | High grade | 35(26.1%) |
| Median follow-up (range) | | 62.2 months (2.57-149.47) |
| Lung cancer-specific death at 10 years | | 81(60.4%) |
