# Supplementary material for: iASPP facilitates tumor growth by promoting mTOR-dependent autophagy in human non-small-cell lung cancer
Source: Cell Death Dis. 2017 Oct 26;8(10):e3150–. doi: 10.1038/cddis.2017.515 (PMC5682680; doi:10.1038/cddis.2017.515)
Supplement: Supplementary Table 2 [file cddis2017515x3.docx]

Table S2 Primer sequences for qRT-PCR

| Name | sequences |
| --- | --- |
| GAPDH -PF: | GACCCCTTCATTGACCTCAAC |
| GAPDH -PR: | CTTCTCCATGGTGGTGAAGA |
| iASPP-PF: | GGCGGTGAAGGAGATGAAC |
| iASPP-PR: | TGATGAGGAAATCCACGATAGAGA |
| OCT4-PF | GACAACAATGAAAATCTTCAGGAGA |
| OCT4-PR | CTGGCGCCGGTTACAGAACCA |
| Nanog-PF: | TGCCTCACACGGAGACTGTC |
| Nanog-PR: | TGCTATTCTTCGGCCAGTTG |
| SOX2-PF: | ACATGAACGGCTGGAGCAAC |
| SOX2-PR: | AGGAAGAGGTAACCACAGGG |
