# Supplementary material for: iASPP facilitates tumor growth by promoting mTOR-dependent autophagy in human non-small-cell lung cancer
Source: Cell Death Dis. 2017 Oct 26;8(10):e3150–. doi: 10.1038/cddis.2017.515 (PMC5682680; doi:10.1038/cddis.2017.515)
Supplement: Supplementary Figure Legends [file cddis2017515x4.docx]

Figure S1. (A) qRT-PCR analysis of the iASPP expression in seven NSCLC cell lines. (B) Western blotting analysis of conversion of LC3-I to LC3-II in knockdown of iASPP in H1299 cells. (C) The altered expression of intracellular signaling proteins in scramble or shiASPP cells was determined using a PathScan® Intracellular Signaling Array. (D) Western blotting analysis of apoptosis-related proteins in scramble or shiASPP cells. The protein expression levels of iASPP in cells were determined by western blotting. GAPDH was used as a loading control. (E) As quality control, Kaplan-Meier survival analysis was performed grouped by lymph node metastasis status or tumor thrombus, which is commonly considered to be associated with survival of patients with lung cancer.
